# Evolution of nMOFs in photodynamic therapy: from porphyrins to chlorins and bacteriochlorins for better efficacy

**DOI:** 10.3389/fphar.2025.1533040

**Published:** 2025-03-18

**Authors:** Yutao Zou, Jiayi Chen, Yijie Qu, Xuanxuan Luo, Weiqi Wang, Xiaohua Zheng

**Affiliations:** ^1^ The People’s Hospital of Danyang, Affiliated Danyang Hospital of Nantong University, Danyang, Jiangsu, China; ^2^ School of Pharmacy, Nantong University, Nantong, Jiangsu, China

**Keywords:** porphyrin, chlorin, bacteriochlorin, metal-organic framework, photodynamic therapy

## Abstract

Photodynamic therapy (PDT) has gained significant attention due to its non-invasive nature, low cost, and ease of operation. Nanoscale metal-organic frameworks (nMOFs) incorporating porphyrins, chlorins, and bacteriochlorins have emerged as one of the most prominent photoactive materials for tumor PDT. These nMOFs could enhance the water solubility, stability and loading efficiency of photosensitizers (PSs). Their highly ordered porous structure facilitates O_2_ diffusion and enhances the generation of ^1^O_2_ from hydrophobic porphyrins, chlorins, and bacteriochlorins, thereby improving their efficacy of phototherapy. This review provides insights into the PDT effects of nMOFs derived from porphyrins, chlorins, and bacteriochlorins. It overviews the design strategies, types of reactive oxygen species (ROS), ROS generation efficiency, and the unique biological processes involved in inhibiting tumor cell proliferation, focusing on the mechanism by which molecular structure leads to enhanced photochemical properties. Finally, the review highlights the new possibilities offered by porphyrins, chlorins, and bacteriochlorins-based nMOFs for tumor PDT, emphasizing how optimized design can further improve the bioapplication of porphyrin derivatives represented PSs. With ongoing research and technological advancements, we anticipate that this review will garner increased attention from scientific researchers toward porphyrin-based nMOFs, thereby elevating their potential as a prominent approach in the treatment of malignant tumors.

## 1 Introduction

Traditional cancer treatments, such as surgery, chemotherapy, and radiotherapy, are effective but have their limitations (Fan et al., 2016; Zhou et al., 2016b; Chen et al., 2019; Xiang et al., 2019). These include the inability to completely eradicate cancer cells, significant side effects, and a high risk of cancer recurrence (Wang et al., 2019b). Phototherapy uses specific wavelengths of light to treat diseases, which has a long history dating back to the 19th century (Hopper, 2000; Ackroyd et al., 2001; Lucky et al., 2015). Phototherapy is minimally invasive and can be repeated without accumulating toxicity, showing promise in improving patients’ quality of life. It is particularly effective in the treatment of superficial bladder cancer and skin cancer (Pham et al., 2021; Chandratre et al., 2023).

The mechanism of PDT relies on the photochemical changes that occur when a PS is activated by LED light or laser (Pandey et al., 1991; O’Connor et al., 2009; Shen et al., 2022; Zhang et al., 2024). After absorbing photons, the PS can transform from its ground state to a singlet excited state (1PS*) (Guo et al., 2024). Subsequently, the excited state can non-radiatively transition to a triplet state (3PS*) (Ma et al., 2024). In this excited state, the photosensitizer initiates two primary types of photochemical reactions: 1) the type I reactions generate ROS such as the superoxide anion (O_2_·^-^), the hydroxyl radical (OH·), and hydrogen peroxide (H_2_O_2_) (Wang et al., 2019c; Chen et al., 2021a; Yu et al., 2022; Wang et al., 2023b; Tang et al., 2024). 2) the type II reactions produce singlet oxygen (^1^O_2_). The strong oxidizing ROS can damage cellular components like proteins, polysaccharides, and lipids, leading to rapid cell death or necrosis (Zheng et al., 2021; Yu et al., 2024).

Due to the good biocompatibility and chemical modifiability, porphyrin-based PSs have been extensively studied in PDT (Bonnett, 1995; Sternberg et al., 1998; Ethirajan et al., 2011; Zou et al., 2024b). From the early use of less pure porphyrin derivatives to the subsequent development of highly purified porphyrin compounds synthesized in laboratories, although significant improvements in purity have been achieved, several challenges remain in their biological applications. Notably, these challenges encompass suboptimal targeting capabilities and insufficient aqueous dispersibility. More critically, these molecules tend to aggregate and precipitate in aqueous environments, such as physiological fluids. This behavior limits their accumulation at tumor sites and affects therapeutic efficacy (Zhang and Yin, 2022; Sun et al., 2023). Therefore, the development of biocompatible and degradable nanocarrier materials represents an effective solution to overcome these challenges.

Over the past three decades, with the rapid advancement of nanotechnology, various types of nanocarrier materials have been developed for the delivery of porphyrin-based PSs in PDT (Zhou et al., 2016a; Rabiee et al., 2020; Tian et al., 2020; Liu et al., 2021; Silva et al., 2021). These nanocarriers can generally be categorized into three classes: 1) organic nanomaterials including dendrimers, micelles (Young et al., 2024), liposomes (Cressey et al., 2022; Jiao et al., 2022; Enzian et al., 2024), and protein nanoparticles (NPs) (Mu et al., 2022; Wang et al., 2022a). 2) inorganic nanomaterials covering porous silica NPs (Prieto-Montero et al., 2023; Nady et al., 2024; Sobhanan et al., 2024), gold NPs, quantum dots (Magaela et al., 2022; Murali et al., 2022; Sangam et al., 2022; Akbar et al., 2023), graphene NPs, and upconversion NPs. (3) hybrid nanomaterials constructed through coordination between inorganic and organic components, such as nMOFs (Ding et al., 2022).

MOFs are a new class of crystalline porous hybrid materials consisting of metal nodes (also known as secondary building units, SBUs) connected by organic linkers (Wang et al., 2012; Zhang and Lin, 2014; Zou et al., 2024c). Due to their periodic structures, high porosity, and excellent biocompatibility, MOFs have become promising drug carriers with broad applications in tumor treatment and bioimaging. Porphyrin-based MOF PSs are of particular interest due to their potential advantages in photodynamic tumor therapy (He et al., 2015; Zou et al., 2024b). These materials efficiently load porphyrin molecules, utilizing their periodically ordered structures to avoid aggregation and increase singlet oxygen generation efficiency. Their porous nature facilitates oxygen transport and ROS diffusion (Lan et al., 2019). The combination of porphyrins with MOFs could effectively enhance solubility and photochemical properties of porphyrin molecules (Park et al., 2015; Liu et al., 2016; Park et al., 2016). Moreover, the specific structure of porphyrin molecules allows them to be reduced via double bonds, yielding derivatives with significantly altered photochemical properties. This has facilitated the development of nMOFs-based photoactive materials with superior performance. For example, the porphyrin molecules can be reduced to chlorin structure, which exhibit increased absorption efficiency in the 640–660 nm wavelength range and enable excellent PDT effect with reduced PSs dose (Lu et al., 2015; Lu et al., 2016). Further reduction to bacteriochlorin structures shifts the maximum absorption wavelength to approximately 740 nm, closer to the near-infrared (NIR) region. This enhancement in light absorption is suitable for improving light penetration in treatments targeting deep-seated tumors (Luo et al., 2020; Xian et al., 2023; Zhang et al., 2023c). Bacteriochlorin molecules can undergo both oxygen-dependent type II photodynamic processes and oxygen-independent type I processes, generating ^1^O_2_, H_2_O_2_, ·OH, and O_2_·^-^, which is crucial for treating hypoxic tumors (Zhang et al., 2023c).

**FIGURE 1:**
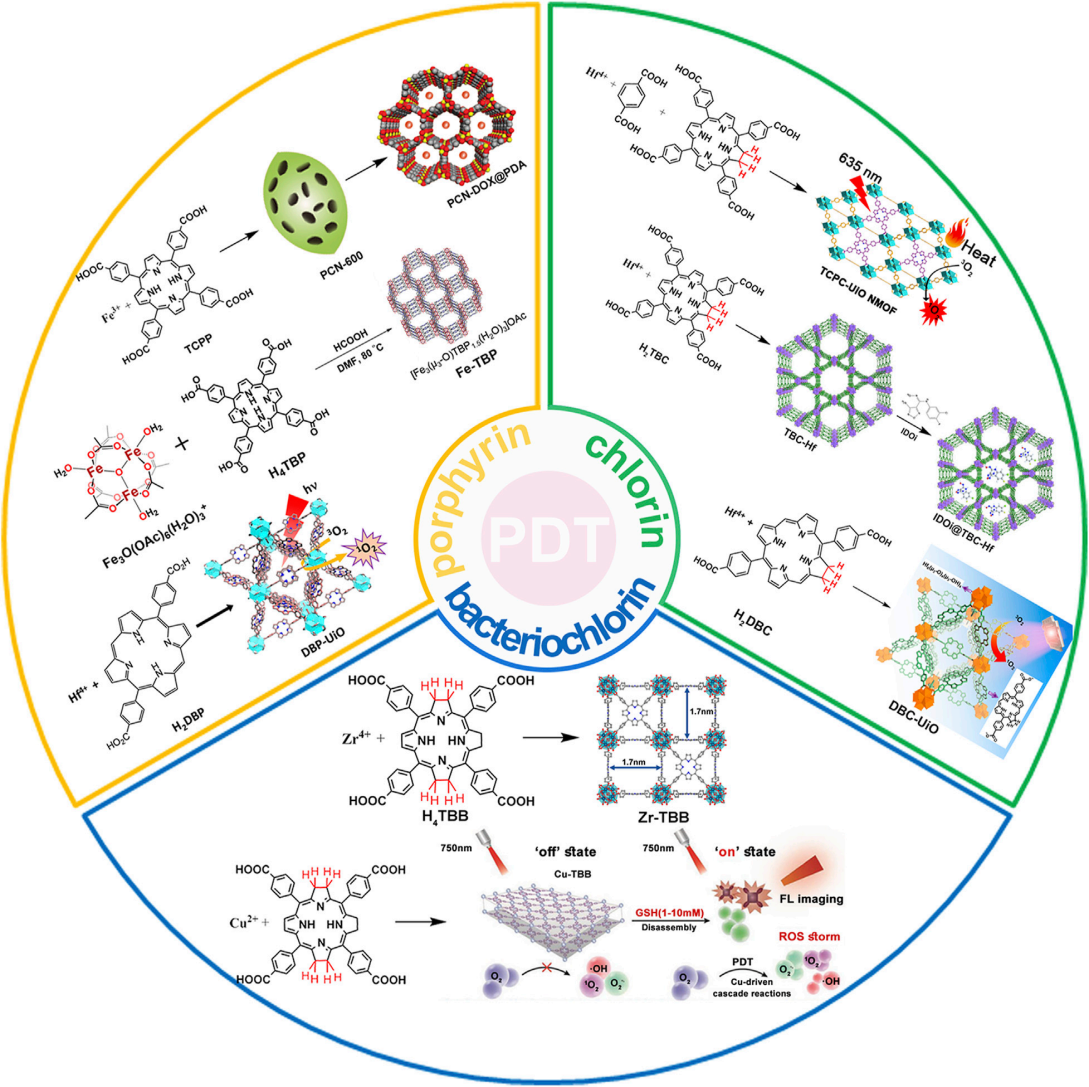
Schematic illustration of the preparation of nMOFs PSs from porphyrin, chlorin and bacteriochlorin. Reprinted with permission. Copyright (2014 (Lu et al., 2014), 2015 (Lu et al., 2015), 2016 (Lu et al., 2016), 2018 (Lan et al., 2018; Zheng et al., 2018), 2020 (Luo et al., 2020), 2023 (Chen et al., 2023b)) American Chemical Society. Copyright 2023 (Yu et al., 2023; Zhang et al., 2023a; Zhang et al., 2023c) John Wiley & Sons, Inc.

This review summarizes the application of porphyrin-, chlorin-, and bacteriochlorin-based nMOFs in photodynamic tumor therapy (Figure 1), focusing on the following aspects: 1) Evolution of molecular structure from porphyrin to chlorin and then bacteriochlorin, progressively optimizing the structure of the PSs. 2) Structural modification enhances excitation light efficiency, increasing the efficiency of light utilization. Especially, modifications to the bacteriochlorin structure enhance light penetration. These changes significantly improve the effectiveness of the light-based treatments. 3) The design of bacteriochlorin introduces type I photodynamic processes, addressing the challenge of treatment in hypoxic environments. In summary, this review highlights recent advancements and limitations of porphyrin MOF materials in PDT applications. In addition, this review aims to advance the biomedical applications of porphyrin-based nMOFs by providing a comparative analysis that spans from concrete examples of applications to the specific molecular mechanisms involved. Through a meticulous analysis and comparison of the molecular structures and photochemical properties of porphyrins, chlorin, and bacteriochlorins, this review endeavors to identify the optimal porphyrin-based photosensitizers. The aim is to develop nanomedicines for highly efficient and low-toxicity photodynamic applications. At last, this review tries to provide valuable insights for future research and development of porphyrin-based nMOFs PSs, thereby advancing their clinical applications.

## 2 Summary of porphyrin-, chlorin- and bacteriochlorin-based nMOFs for efficient PDT

After synthesizing the porphyrin derivatives in the laboratory, we can confirm its structure using ^1^H NMR and mass spectrometry. Then we can employ solvothermal methods to prepare MOF materials. Specifically, this involves dissolving metal salts and organic ligands in a solvent. The resulting mixture is then reacted in an autoclave under specific temperatures and autogenous pressures to yield the desired product. Combining metals such as Zr, Hf, Fe, Cu ions with porphyrins, chlorins, and bacteriochlorin, the nanomaterials can be produced along with an appropriate amount of acid as a catalyst. Via controlling reaction times and different amounts of ligands, we can get the nMOFs with sizes less than 200 nm, maintaining high crystallinity and good dispersion. These materials exhibit efficient photochemical reactions under weak LED or laser irradiation. This process generates highly reactive oxygen species, which can be harnessed for PDT. Additionally, the porous nature of nMOFs allows for the combination of multifunctional treatment modalities, effectively inhibiting hypoxic or metastatic tumors (Table 1). This review summarizes recent advancements in the coordination of representative porphyrin molecules, chlorins, and bacteriochlorins with metals. This coordination produces nMOFs with varying sizes, photoreactivity, and therapeutic efficacies for the eradication of malignant tumors through PDT (Table 1).

**TABLE 1:** Porphyrin, chlorin, and bacteriochlorin-based nMOFs for PDT applications.

Material	Structure		Function	References
	Porphyrin	Nanoscale MOFs		
Hf DBP-UiO	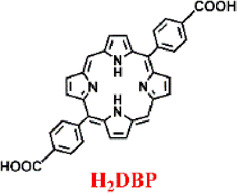	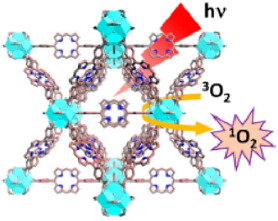	100 nm, nanoplate, 640 nm, 180 J/cm^2^, PDT	Lu et al. (2014)
Fe-TCPP	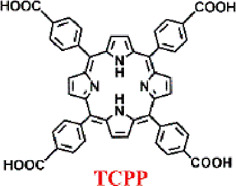	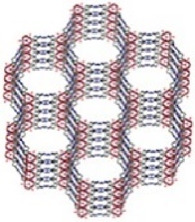	100 nm, nanorice, 650 nm, 45 J/cm^2^, PDT+ICD	Lan et al. (2018)
Fe-TCPP	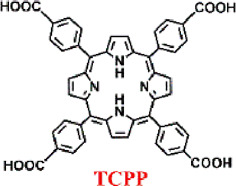	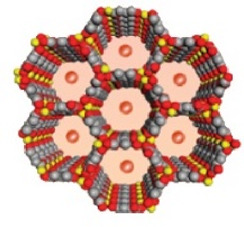	180 nm, spindle, 633 nm, 808 nm PDT+PTT	Chen et al. (2023b)
Fe-TCPP	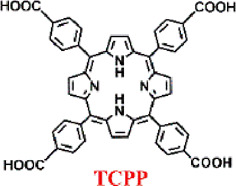	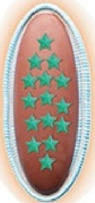	50 nm–150 nm, 670 nm, PDT+Chemo+Ferro	Zhang et al. (2023a)
Hf DBC-UiO	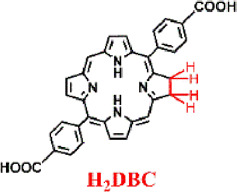	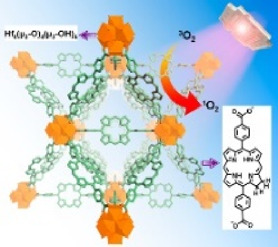	100–200 nm, nanoplate 650 nm, 90 J/cm^2^, PDT	Lu et al. (2015)
Hf-TCPC	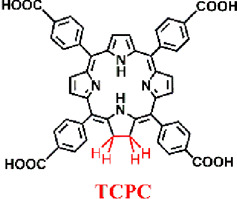	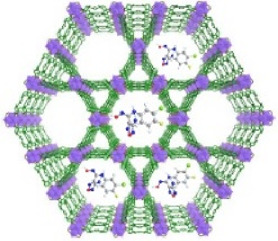	50–100 nm long, nanorice, 650 nm, 90 J/cm^2^, PDT+Immuno	Lu et al. (2016)
Hf TCPC-UiO	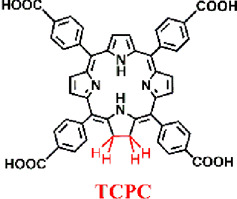	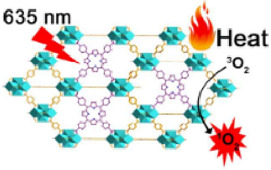	100–130 nm, 21.6 nm, 635 nm, PDT+PTT	Zheng et al. (2018)
Zr-TBB	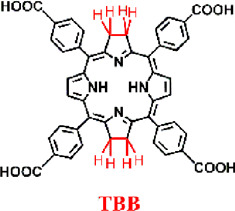	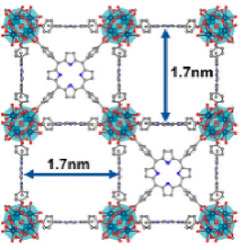	100 nm, 740 nm, Type I, II PDT	Luo et al. (2020)
Cu-TBB	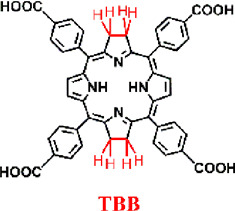	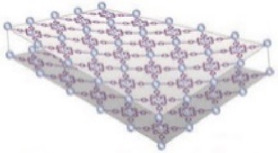	50–200 nm, nanosheet 750 nm, Type I, II PDT + Pyro	Zhang et al. (2023c)

Abbreviated specification: (ICD, immunogenic cell death; PTT, photothermal therapy; Chemo, chemotherapy, Ferro, ferroptosis; Immuno, immunotherapy; ST, Starvation therapy; Pyro, pyroptosis).

## 3 Porphyrin-based nMOFs for photodynamic therapy

As an efficacious modality for cancer treatment, the PDT method hinges on the synergy of three pivotal components: photosensitizers (PSs), suitable light sources, and oxygen (Fan et al., 2016; Guo et al., 2023; Zhu et al., 2024; Zou J. et al., 2024). To enhance the clinical efficacy of PDT, researchers have been dedicated to optimizing these key elements. An ideal photosensitizer should adhere to stringent criteria: clear structure, facile and cost-effective synthesis, excellent photochemical stability, water solubility for efficient administration, tumor-targeting capability to minimize damage to non-targeted regions, and low dark toxicity (Guo et al., 2023). As technology advances, photosensitizers have evolved from first-generation to fourth-generation versions, with the development of the latter particularly highlighting the potential of inorganic-organic hybrid materials such nMOFs (He et al., 2015). Various research teams have pioneered the design of fourth-generation photosensitizers through the loading of PS molecules onto nMOFs (Wang et al., 2022b). Owing to the unique nanoscale features and inherent MOF properties, nMOFs have demonstrated significant advantages in biomedical applications (Ni et al., 2018; Luo et al., 2021). Their high porosity enables efficient drug loading. Functional surface groups allow nMOFs to integrate multiple functionalities, facilitating precise drug delivery and release, which is critical for combination therapies and diagnostic imaging (He et al., 2015). Additionally, the biodegradability of nMOFs ensures controlled drug release within the tumor microenvironment.

**FIGURE 2:**
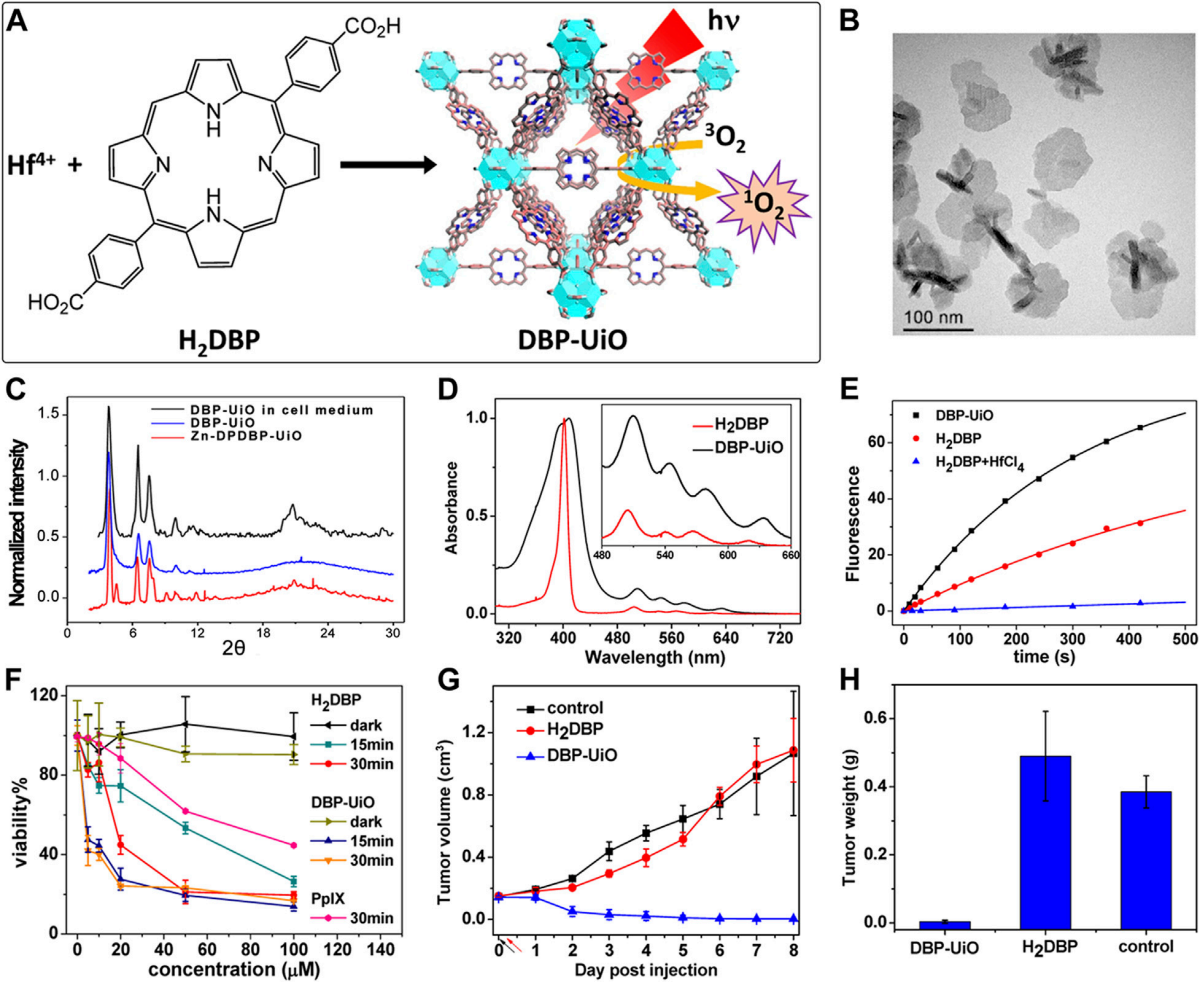
**(A)** Preparation of Hf-DBP nMOF. **(B)** TEM image of DBP-UiO exhibiting a nanoplate structure. **(C)** PXRD patterns of Zn-DPDBP-UiO, DBP-UiO, and DBP-UiO following 12 h of incubation in RPMI 1640 cell culture medium. **(D)** Absorbance spectra of H_2_DBP and DBP-UiO in PBS. **(E)** ^1^O_2_ production by DBP-UiO, H_2_DBP, and H_2_DBP in combination with HfCl_4_. **(F)** *In vitro* PDT cytotoxicity of H_2_DBP, DBP-UiO, and protoporphyrin IX (PpIX) at varying PS concentrations and exposure times. **(G)** Tumor growth inhibition profile following PDT treatment. **(H)** Tumor weight post-PDT across experimental groups. Reprinted with permission. Copyright 2014 American Chemical Society (Lu et al., 2014).

Acting as carriers for PSs, MOFs can effectively prevent self-quenching due to the aggregation of PS molecules (Lu et al., 2018). Simultaneously, their porous structure facilitates the rapid diffusion of ROS, enhancing the cytotoxicity against cancer cells. In 2014, Lin et al. reported for the first time the UiO topology-based DBP-UiO nanocrystals formed by the coordination of metal Hf with dicarboxylic acid porphyrin ligands, which were nanoplate-shaped in geometry, for *in vivo* PDT (Figures 2A, B) (Lu et al., 2014). Powder X-ray diffraction (PXRD) confirmed that DBP-UiO adopted a UiO-type MOF structure (Figure 2C). Subsequent UV-visible spectroscopy showed that H_2_DBP displayed a Soret band at 402 nm and four Q bands at 505, 540, 566, and 619 nm. All Q bands of DBP-UiO were slightly red-shifted, peaking at 510, 544, 579, and 634 nm, which may be attributed to the coordination of the carboxylate group of the DBP ligand with the Hf^4+^ center. The presence of four Q bands and their red-shift further supports the existence of radical porphyrin ligands in DBP-UiO (Figure 2D). Under illumination by a 640 nm LED light source, the chemiluminescent reagent SOSG reacted with ^1^O_2_ to generate green fluorescence. This fluorescence was quantified, showing that DBP-UiO generates ^1^O_2_ at least twice as efficiently as H_2_DBP. This increased efficiency may be due to the heavy Hf^4+^ center, which facilitates intersystem crossing from singlet to triplet excited states of DBP (Figure 2E). Given its superior singlet oxygen production efficiency, the authors preceded with cytotoxicity and animal tumor suppression experiments. They chose human head and neck cancer cells SQ20B, which are resistant to cisplatin and radiotherapy, for PDT (Figure 2F). Notably, the group treated with DBP-UiO exhibited significantly enhanced PDT efficacy compared to other control groups, even at a PS dose of 5 μM and a 15 min irradiation period (Figure 2F). Subsequently, *in vivo* experiments on SQ20B subcutaneous xenograft mouse models revealed that mice treated with DBP-UiO began showing tumor shrinkage 1 day after the DBP-UiO administration and PDT, demonstrating favorable antitumor effects (Figures 2G, H). This work underscores how the fusion of porphyrin molecules with NMOFs modulates their photophysical and photochemistry properties. It also highlights the emergence of a new class of highly effective PDT agents. These agents hold promise for clinical application against cancers resistant to chemotherapy and radiotherapy.

**FIGURE 3:**
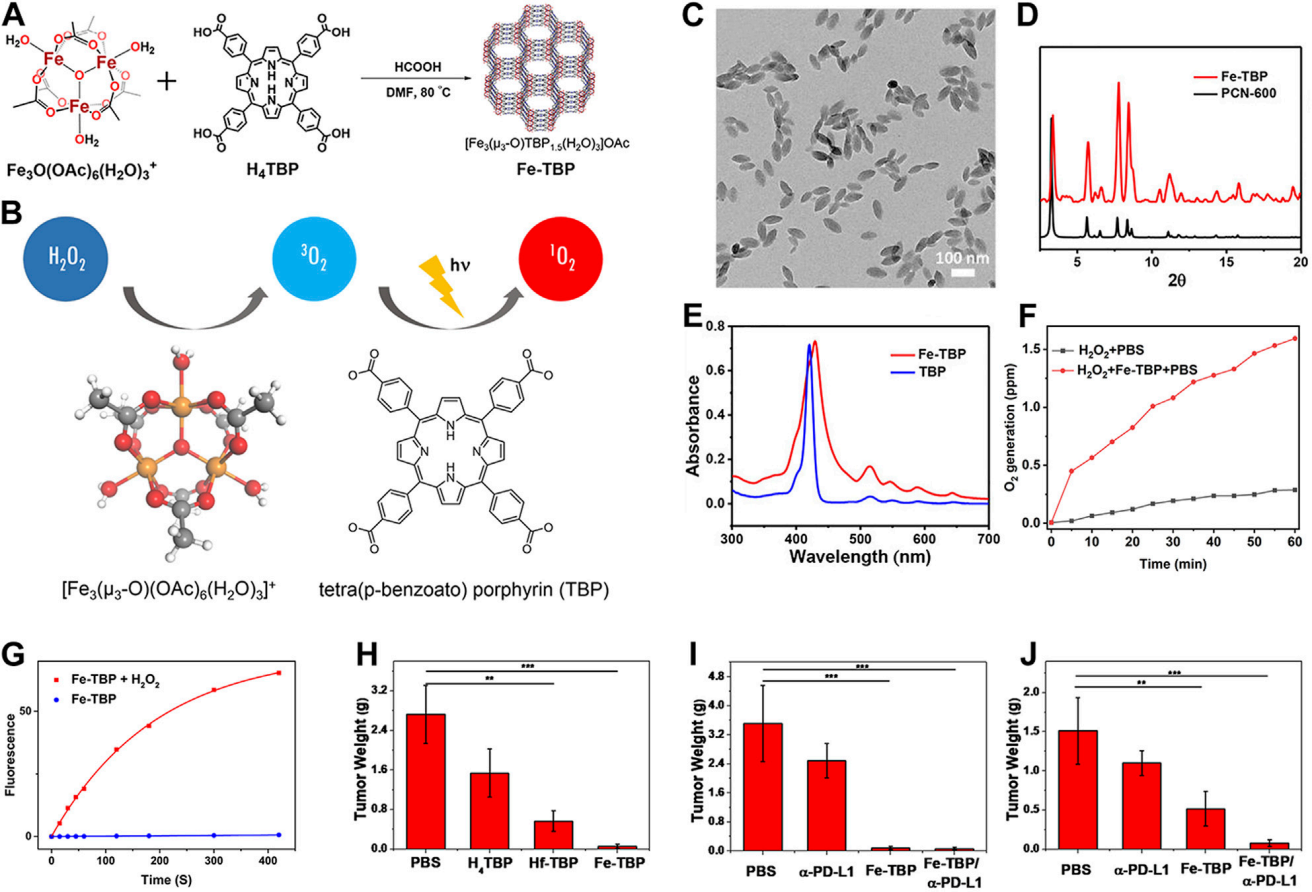
**(A)** Synthesis route of Fe-TBP and **(B)** cascade reaction of Fe-TBP to transfer H_2_O_2_ into ^1^O_2_ under hypoxic condition. **(C)** TEM image of optimized 100 nm Fe-TBP. **(D)** PXRD pattern of Fe-TBP in comparison to PCN-600. **(E)** UV-visible spectra of Fe-TBP and H_4_TBP. **(F)** Time-dependent O_2_ generation detected by an oxygen sensor. **(G)** ^1^O_2_ generation of Fe-TBP with or without H_2_O_2_ in oxygen-free DMF solution detected by SOSG assay. **(H)** Tumor weights from each treatment group on a CT26 single tumor model after. Tumor weights **(I)** of primary tumors from each treatment group on a CT26 bilateral tumor model. Tumor weights **(J)** of distant tumors from each treatment group on a CT26 bilateral tumor model. Reprinted with permission. Copyright 2018 American Chemical Society (Lan et al., 2018).

Despite the significant enhancement of PDT efficacy by nMOFs, hypoxic solid tumors remain a limiting factor for this therapeutic approach. To address this issue, the Lin research group developed an oxygen-self-supplying integrated MOF system. This system modifies the tumor microenvironment through PDT-induced acute inflammatory responses. It also potentiates the effectiveness of immune checkpoint blockade (ICB). As a result, it achieves immunogenic PDT that overcomes tumor hypoxia (Lan et al., 2018). Constructed from iron-oxide clusters and porphyrin ligands, Fe-TBP (Figure 3A) catalyzes a series of reactions under hypoxic conditions upon irradiation. Intracellular H_2_O_2_ is decomposed into O_2_ via Fenton-like reactions catalyzed by iron-oxide clusters, and the generated O_2_ is converted into cytotoxic ^1^O_2_ by the light-excited porphyrin (Figure 3B). TEM confirmed the nanoparticle morphology of Fe-TBP (Figure 3C). PXRD result indicated that Fe-TBP exhibits a PCN-600 structure (Figure 3D). UV-vis spectroscopy evidenced successful coordination of the tetracarboxylic porphyrin (Figure 3E). Oxygen sensor measurements revealed an increase in O_2_ concentration over time in the presence of H_2_O_2_, suggesting Fe-TBP’s potential to ameliorate the hypoxic tumor microenvironment and augment PDT efficacy (Figure 3F). Moreover, under both illuminated and hypoxic conditions following the addition of H_2_O_2_, Fe-TBP was capable of generating ^1^O_2_, confirming the effectiveness of iron ion-catalyzed oxygen production (Figure 3G). Subsequently, animal experiments on CT26 tumor-bearing mice demonstrated the unilateral tumor model’s proof-of-concept for the efficacy of Fe-TBP in suppressing tumor growth (Figure 3H). Bilateral tumor models also confirmed that Fe-TBP-mediated PDT enhanced the efficacy of ICB and induced bystander effects (Figures 3I, J). This innovative porphyrin nMOF successfully overcomes tumor hypoxia in PDT and improves cancer immunotherapy, marking a significant step forward in the field. Immunotherapy is recognized by many researchers as an effective treatment method for cancer (Chen and Wang, 2020; Hu et al., 2020b; Mao et al., 2023). This novel strategy in this work not only addresses the critical challenge of tumor hypoxia in PDT but also synergizes with immunotherapy, offering a promising avenue for the treatment of solid tumors. By leveraging the oxygen-supplying capacity of Fe-TBP and the tumor-modifying effects of PDT, the Lin group has paved the way for more effective cancer therapy that can harness the patient’s own immune system to combat the disease. The integration of PDT with ICB represents a breakthrough in the fight against solid tumors, particularly those characterized by low oxygen levels that have historically posed a significant barrier to successful treatment.

**FIGURE 4:**
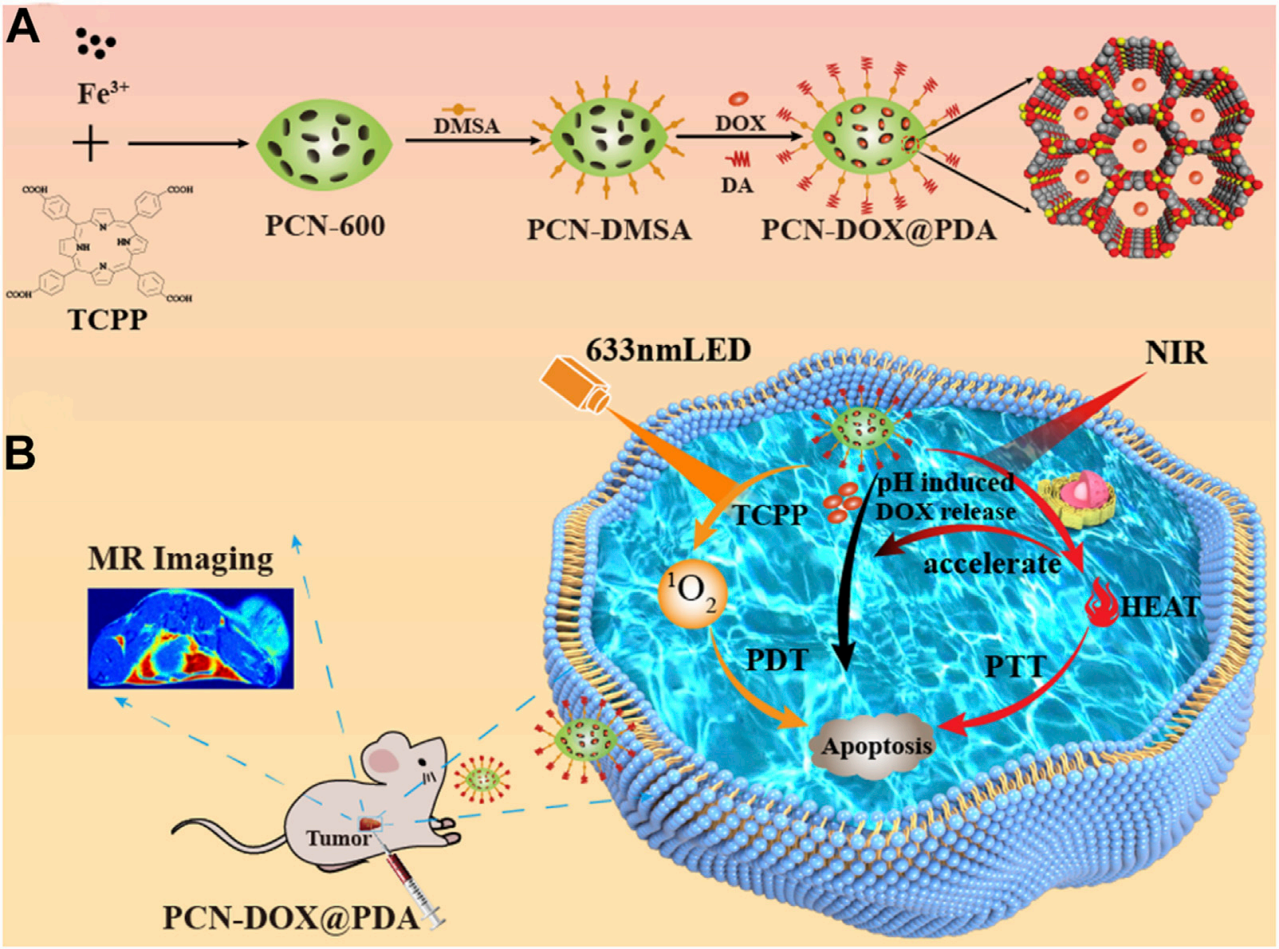
**(A)** Preparation of PCN-DOX@PDA NPs. **(B)** Cytotoxic mechanism employed by PCN-DOX@PDA against malignant cells. Reprinted with permission. Copyright 2023 American Chemical Society (Chen et al., 2023b).

The porous nature of nMOFs allows their channels to be used for loading chemotherapeutic drug molecules. Through a core-shell structure and surface modification with polydopamine (PDA), these materials can achieve a triple-combined therapeutic effect, including photodynamic therapy, chemotherapy, and photothermal therapy (Chen et al., 2023b). For instance, Chen et al. employed Fe^3+^ coordinated with TCPP to form PCN-600, which was loaded with the chemotherapeutic agent doxorubicin (DOX) and further modified with a polydopamine shell, creating the PCN-DOX@PDA nanoplatform (Chen et al., 2023b). PCN-DOX@PDA is capable of effectively producing ^1^O_2_, and the presence of PDA enhances NIR light absorption, leading to a strong photothermal effect in PCN-DOX@PDA NPs. The weakly acidic environment of the tumor microenvironment degrades PCN-DOX@PDA, thereby releasing the antitumor drug DOX. The heating effect of photothermal therapy accelerates this drug release thus inhibiting tumor cell growth (Figures 4A, B). This conceptual framework underscores the potential of porphyrin-based MOFs for clinical applications, particularly their ability to achieve multimodal therapy through surface modifications. Strategies such as introducing folic acid or small molecule peptides can significantly enhance the tumor specificity and biomedical applicability of MOFs. Through these surface engineering approaches; the performance of NPs is enhanced. This results in more potent anticancer activities in both cellular and animal models.

**FIGURE 5:**
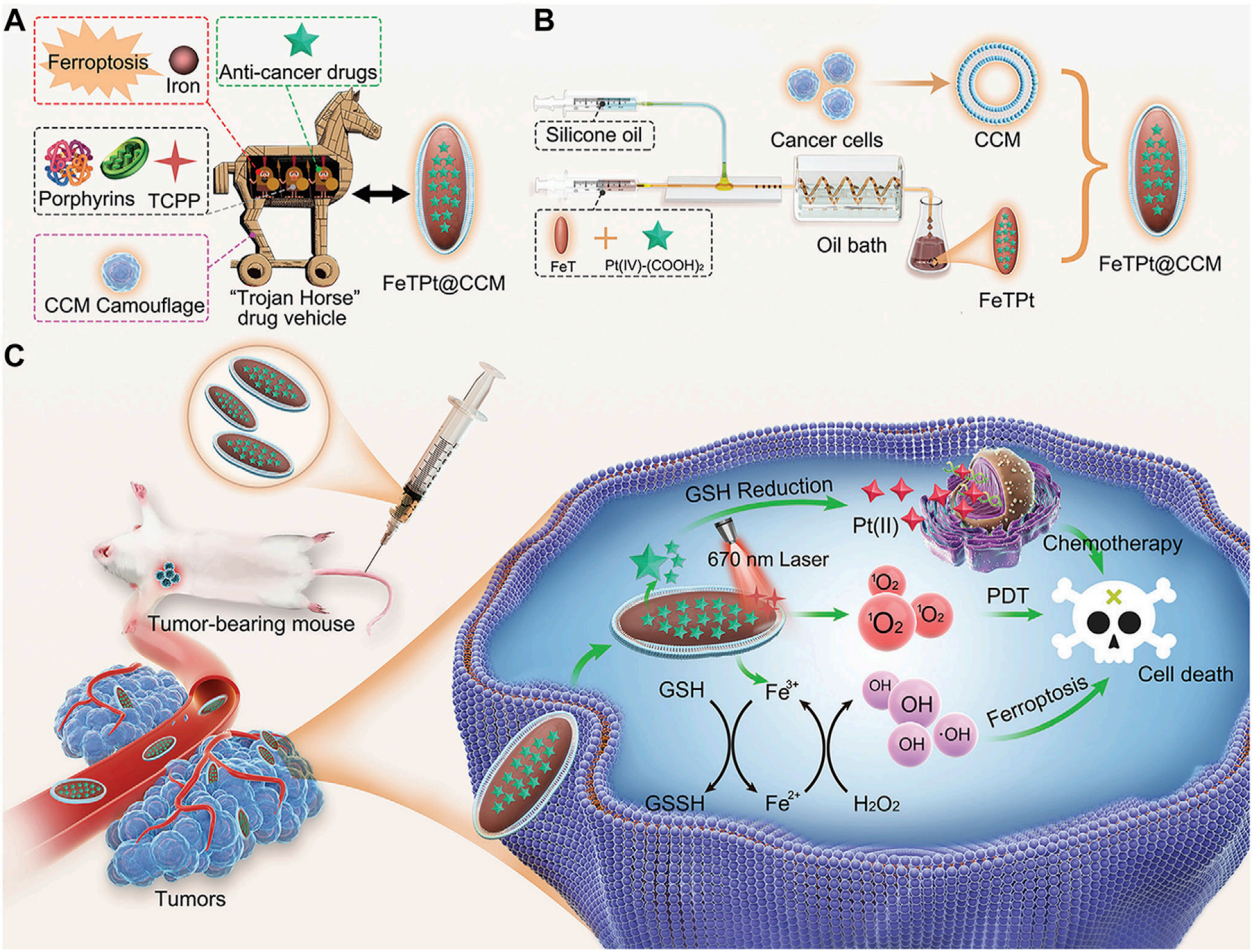
**(A)** Conceptualization of a multi-functional “Trojan Horse” nanovehicle for drug delivery. **(B)** Development of FeTPt via microfluidic synthesis, followed by encapsulation with CCM to form FeTPt@CCM. **(C)** FeTPt@CCM exerts the combination therapeutic effect of chemotherapy, ferroptosis induction, and PDT. Reprinted with permission. Copyright 2023 John Wiley & Sons, Inc (Zhang et al., 2023a).

Despite the significant therapeutic effects of porphyrin-based nMOFs in phototherapy, these nanosystems still face challenges such as immune clearance and poor cancer cell targeting. Researchers have discovered that encapsulating nMOFs with a cell membrane, a top-down biomimetic strategy, can reduce their phagocytosis by macrophages. This encapsulation also significantly enhances the homologous targeting ability of nMOFs. The integration of cell membranes with nMOFs represents a promising approach to further improve the preclinical applications of these materials. Combining the unique properties of nMOFs with the natural functionalities of cell membranes may lead to more effective and targeted therapies (Zhang et al., 2023a). For example, Zhang et al. engineered an innovative MOF nanosystem incorporating Fe^3+^, TCPP, and the prodrug precursor oxaliplatin, designated as FeTPt (Zhang et al., 2023a). To augment its stealth and affinity, the nanosystem was enveloped in a biomembrane derived from cancer cell membranes (CCMs), creating a Trojan horse-like delivery system (FeTPt@CCM). Bearing CCM components on its surface, FeTPt@CCM adeptly identifies and accumulates in tumor tissues, exploiting its homing binding mechanism (Figures 5A, B). Upon phagocytosis by tumor cells, it catalyzes the production of hydroxyl radicals and oxygen through a process akin to the Fenton reaction, as well as the redox interactions between Fe^3+^ and intracellular glutathione (GSH) and H_2_O_2_. This cascade triggers ferroptosis pathways while simultaneously amplifying the potency of PDT. Concurrently, oxaliplatin, as a platinum-based chemotherapeutic, synergistically reinforces these effects, collectively suppressing the proliferation of cancer cells and tumor progression (Figure 5C). In summary, the FeTPt@CCM nanoplatform achieves a triple synergistic impact with its distinctive structural design. This platform combines PDT, ferroptosis induction, and chemotherapy. It offers a novel, efficient, and precise strategy for cancer treatment. This achievement not only illuminates the tremendous potential of porphyrin-based MOFs in oncology but also underscores the pivotal role of nanotechnology in enhancing drug delivery efficacy and therapeutic outcomes. This breakthrough showcases the versatility of MOFs in biomedical applications. Specifically in cancer therapy, their customizable properties can address specific therapeutic challenges. The integration of biomimetic membranes boosts the targeting precision of MOFs. It also confers additional functionalities, such as immune evasion and homing to diseased sites. These enhancements make MOFs formidable platforms for advanced drug delivery systems. This multidisciplinary approach merges the strengths of materials science with biomedicine. It paves the way for the development of sophisticated therapeutics that can be tailored to individual patient needs. This advancement heralds a new era in personalized medicine. The promising results from preclinical studies like this one offer hope for the future clinical translation of these technologies, potentially transforming the landscape of cancer treatment.

## 4 Chlorin-based nMOFs for photodynamic therapy

**FIGURE 6:**
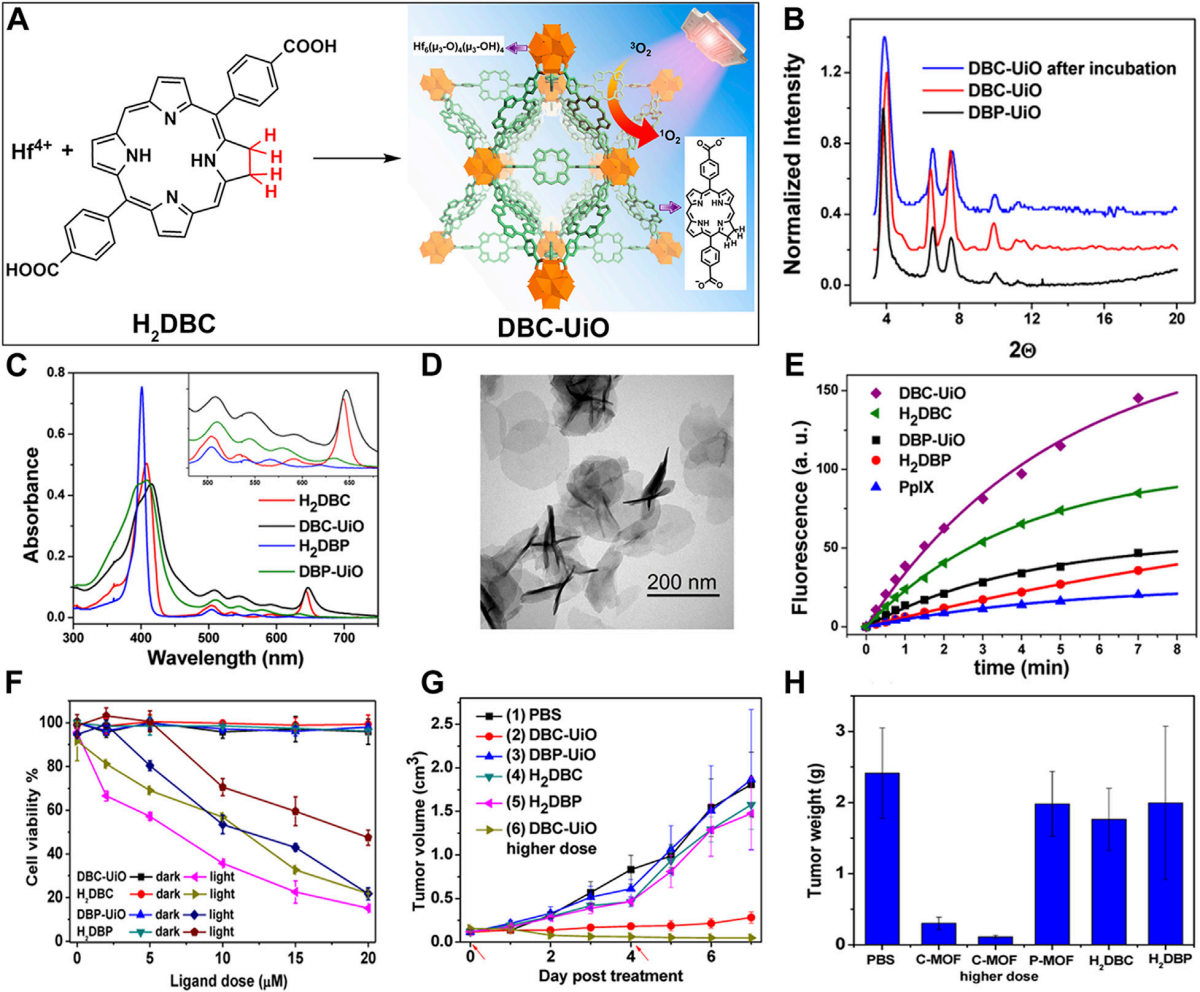
**(A)** Preparation of DBC-UiO. **(B)** PXRD analysis of DBP-UiO and DBC-UiO. **(C)** Comparative UV-visible absorption spectroscopy of H_2_DBC, DBC-UiO, H_2_DBP, and DBP-UiO in DMF and PBS solutions at a concentration of 0.67 mM. **(D)** TEM imagery of DBC-UiO demonstrating nanoplate configuration prior to cell culture medium incubation. **(E)** Quantification of ^1^O_2_ yield from DBC-UiO, H_2_DBC, DBP-UiO, H_2_DBP, and PpIX under LED irradiance of 0.1 W/cm^2^, with DBC-UiO and H_2_DBC illuminated at 650 nm, and others at 640 nm. **(F)** PDT efficacy on CT26 cells as indicated by cytotoxicity levels at various PS concentrations for DBC-UiO, DBP-UiO, H_2_DBC, and H_2_DBP. **(G)** Tumor growth suppression in CT26 tumor models following PDT interventions. **(H)** Tumor masses in CT26 models post-PDT treatment. Reprinted with permission. Copyright 2015 American Chemical Society (Lu et al., 2015).

Porphyrins nMOFs-based PDT has been utilized in the treatment of cancers and other diseases due to its ability to minimize collateral damage to normal tissues compared to systemic therapies (Li et al., 2017; Wang et al., 2019a; Hao et al., 2021; Wu et al., 2022; Zhao et al., 2023). However, the photophysical properties of porphyrin-based MOFs are suboptimal, with a decreasing intensity from characteristic to non-characteristic absorption peaks and the lowest energy absorption typically around 640 nm. At the maximum absorption wavelength, the molar extinction coefficients are relatively low, which is disadvantageous for the excitation of porphyrins and their derivatives using red light in the 630–650 nm range for PDT. Addressing this limitation, Lu et al. coordinated Hf^4+^ with dicarboxylate chlorin (H_2_DBC) to synthesize DBC-UiO, which exhibited superior photophysical characteristics over previously reported DBP-UiO (Figure 6A) (Lu et al., 2015). PXRD analysis confirmed the UiO-type crystalline structure of DBC-UiO, and it displayed good stability in cell culture media (Figure 6B). UV-visible spectroscopy revealed the chemical reduction of the porphyrin ligand in DBP-UiO to form the chlorin ligand in DBC-UiO. This transformation led to significant spectral shifts, particularly a 13 nm red shift in the lowest-energy Q-band and an 11-fold increase in the extinction coefficient at this band (Figure 6C). TEM observations showed that DBC-UiO had nanosheet morphology with dimensions ranging from 100 to 200 nm (Figure 6D). Using SOSG as a ROS detection probe, which fluoresces green (emission at 525 nm) upon reaction with generated ^1^O_2_, quantitative fluorescence measurements indicated that DBC-UiO produced ^1^O_2_ approximately three times more efficiently than DBP-UiO (Figure 6E). Leveraging this enhanced ^1^O_2_ generation capacity, the authors conducted *in vitro* and *in vivo* tumor suppression experiments. After incubating CT26 cells with either DBP-UiO or DBC-UiO and subsequent illumination with LED light (DBP-UiO and H_2_DBP, 640 nm; DBC-UiO and H_2_DBP, 650 nm), with a total light dose of 90 J/cm^2^ (0.1 W/cm^2^, 15 min), cell viability assays showed that DBC-UiO effectively eradicated cancer cells at lower nMOF and light doses, outperforming DBP-UiO (Figure 6F). Subsequently, in a mouse model with subcutaneously implanted CT26 tumors, administration of the photosensitizers followed by light therapy showed that DBC-UiO was more efficacious than DBP-UiO. This was evidenced by a greater reduction in tumor volume and weight (Figures 6G, H).

These findings underscore the immense potential of chlorin-based nMOFs as a superior nanomaterial platform. They also highlight these nMOFs’ exceptional PDT efficacy at the cellular level, foreshadowing broad application prospects in biomedicine. More importantly, when the perspective is extended to animal models, similarly impressive PDT performance is observed. This marks a critical step toward translating laboratory successes into clinical applications. Consequently, chlorin-based nMOFs represent not only a theoretical innovation in nanodrug design but also demonstrate practical excellence. This signals substantial translational potential in cancer therapy and positions them as a promising component of future precision medicine and personalized treatment strategies.

The localized nature of light exposure in PDT renders it ineffective against disseminated diseases (Zeng et al., 2018; Hu et al., 2020a). As such, there is a need for more potent PSs and novel therapeutic strategies to enhance PDT’s efficacy in eradicating local tumors and controlling distant metastases. Immunotherapy has garnered attention as a revolutionary approach in cancer treatment. It has achieved high overall response rates across various cancer types. Additionally, it has provided long-term tumor control in some patients (Ni et al., 2020). Significantly, checkpoint blockade immunotherapy stands out by modulating the dysregulated expression and function of immune checkpoint proteins through small molecules or antibodies, reactivating the immune environment suppressed by tumors. As an immunoregulatory enzyme, Indoleamine 2,3-dioxygenase (IDO) is excessively active within tumors. It catalyzes the conversion of tryptophan to kynurenine. IDO has become a focal point in this domain (Dai et al., 2022; Fujiwara et al., 2022; Zhang et al., 2022; Wang et al., 2023a). Research combining IDO inhibitors (IDOi) is currently drawing considerable interest from the scientific community. The porous nature of chlorin-based nMOFs makes them effective carriers for IDOi.

**FIGURE 7:**
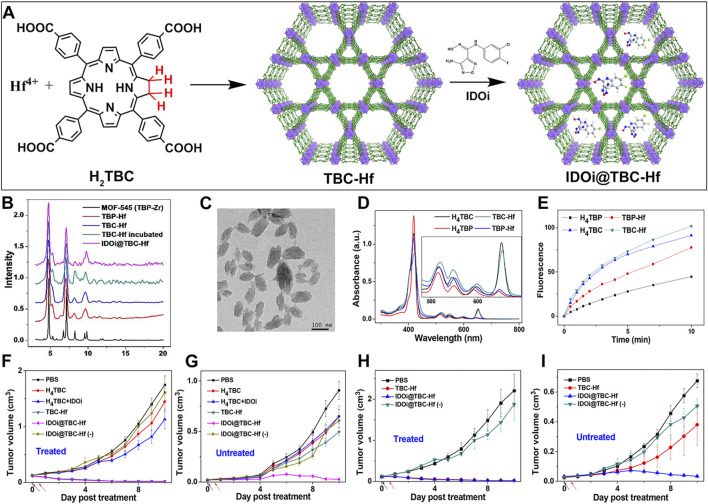
**(A)** Preparation procedures of TBC-Hf and IDOi@TBC-Hf. **(B)** Comparison of the PXRD profiles between TBP-Hf, TBC-Hf, and IDOi@TBC-Hf, juxtaposed with those of MOF-545. **(C)** TEM visuals of the TBC-Hf structure **(D)** Spectroscopic analysis displaying the UV-visible absorption characteristics of H_4_TBP, H_4_TBC, TBP-Hf, and TBC-Hf. An inset provides an enhanced view focusing on the Q-band region. **(E)** Measurement of ^1^O_2_ production by H_4_TBP, H_4_TBC, TBP-Hf, and TBC-Hf carried out using the Singlet Oxygen SensorGreen assay. **(F)** Illustration of the tumor growth trajectory in treated CT26 tumor-bearing mice following PDT. **(G)** Representation of the growth kinetics for untreated tumors in CT26 tumor-bearing mice post-PDT treatment. **(H)** Charting the growth dynamics of treated MC38 tumor-bearing mice after undergoing PDT. **(I)** Tracking the growth pattern of untreated tumors in MC38 tumor-bearing mice subsequent to PDT. Reprinted with permission. Copyright 2016 American Chemical Society (Lu et al., 2016).

Building on this premise, Lu et al. synthesized a chlorin-based nMOF (TBC-Hf) by coordinating tetracarboxylic chlorin with Hf^4+^, and leveraged the highly porous structure of nMOFs to load IDOi into TBC-Hf, resulting in IDOi@TBC-Hf (Figure 7A) (Lu et al., 2016). PXRD indicated that the structure of TBC-Hf resembled that of MOF-545 (Figure 7B) and remained stable in cell culture media even after loading with IDOi. TEM analysis revealed the nanoparticle morphology of TBC-Hf (Figure 7C). UV-visible absorption spectra showed that TBC-Hf absorbed red light more effectively than TBP-Hf (a porphyrin-based nMOF) (Figure 7D). H_4_TBC exhibited a pronounced absorption peak at 420 nm, with four additional peaks between 518 and 652 nm. TBC-Hf had its main peak at 421 nm, with Q-bands spanning from 520 to 653 nm. The molar extinction coefficient of H_4_TBC at its maximum absorption zone was nine times greater than that of H_4_TBP; whereas TBC-Hf was 6 times higher than TBP-Hf (Figure 7D). ^1^O_2_ generation tests using the SOSG probe demonstrated that TBC-Hf surpassed both TBP-Hf and H_4_TBC in its efficiency (Figure 7E). The systemic effects of combining IDOi with PDT using TBC-Hf were evaluated in immunocompetent rodent models featuring bilateral tumor models of CT26 colorectal cancer (BALB/c mice) and MC38 colorectal cancer (C57BL/6 mice). One tumor received intratumoral injections of IDOi@TBC-Hf followed by light exposure, designated as the “treated tumor,” while the contralateral tumor remained untreated. It was found (Figures 7F, H) that local injection of nMOF and light exposure significantly inhibited the growth of the treated tumor. Notably, the PDT effect of IDOi@TBC-Hf was observed to reduce the size of the contralateral untreated tumor exclusively in the treated group (Figures 7G, I). In both the CT26 and MC38 models, the untreated tumors began to shrink starting on day 6 and day 5 post-treatment, respectively, indicating the induction of a systemic antitumor immune response. This confirms that the combination of local PDT with immune checkpoint inhibition enhances control over distant tumors. Combining chlorin-based nMOFs with IDOi optimizes the synergy between local treatment efficacy and the stimulation of a comprehensive immune response. This approach effectively curtails the progression of both primary and distant tumors. It also minimizes adverse effects, opening new avenues for the integrated treatment of metastatic colorectal cancer.

Integrative diagnostics and therapeutics combine diagnostic and therapeutic functions on a single platform, enabling real-time detection of disease and targeted treatment. This also allows for monitoring drug distribution in the body, significantly enhancing medical efficiency and precision. The synergistic combination of photothermal and photodynamic therapies leverages complementary treatment modalities. This combination can exert synergistic effects against various tumor cell types, markedly improving therapeutic outcomes. Photothermal therapy is utilized as either a standalone treatment or as one of the combination therapies for the treatment of various diseases, particularly cancer (Su et al., 2019; Yu et al., 2019; Chen et al., 2021b; Liu et al., 2022). The integration of PDT with PTT uses the photothermal effect to disrupt the physical structure of tumors. It also employs photodynamic action to induce cytotoxicity, overcoming the limitations of monotherapies and intensifying antitumor activity while reducing damage to surrounding healthy tissues (Overchuk et al., 2023; Wang et al., 2024). Incorporating multimodal imaging and PTT into a porphyrin-based nMOFs system, which is typically limited to PDT efficacy, may augment therapeutic potency and precision (Zheng et al., 2018).

**FIGURE 8:**
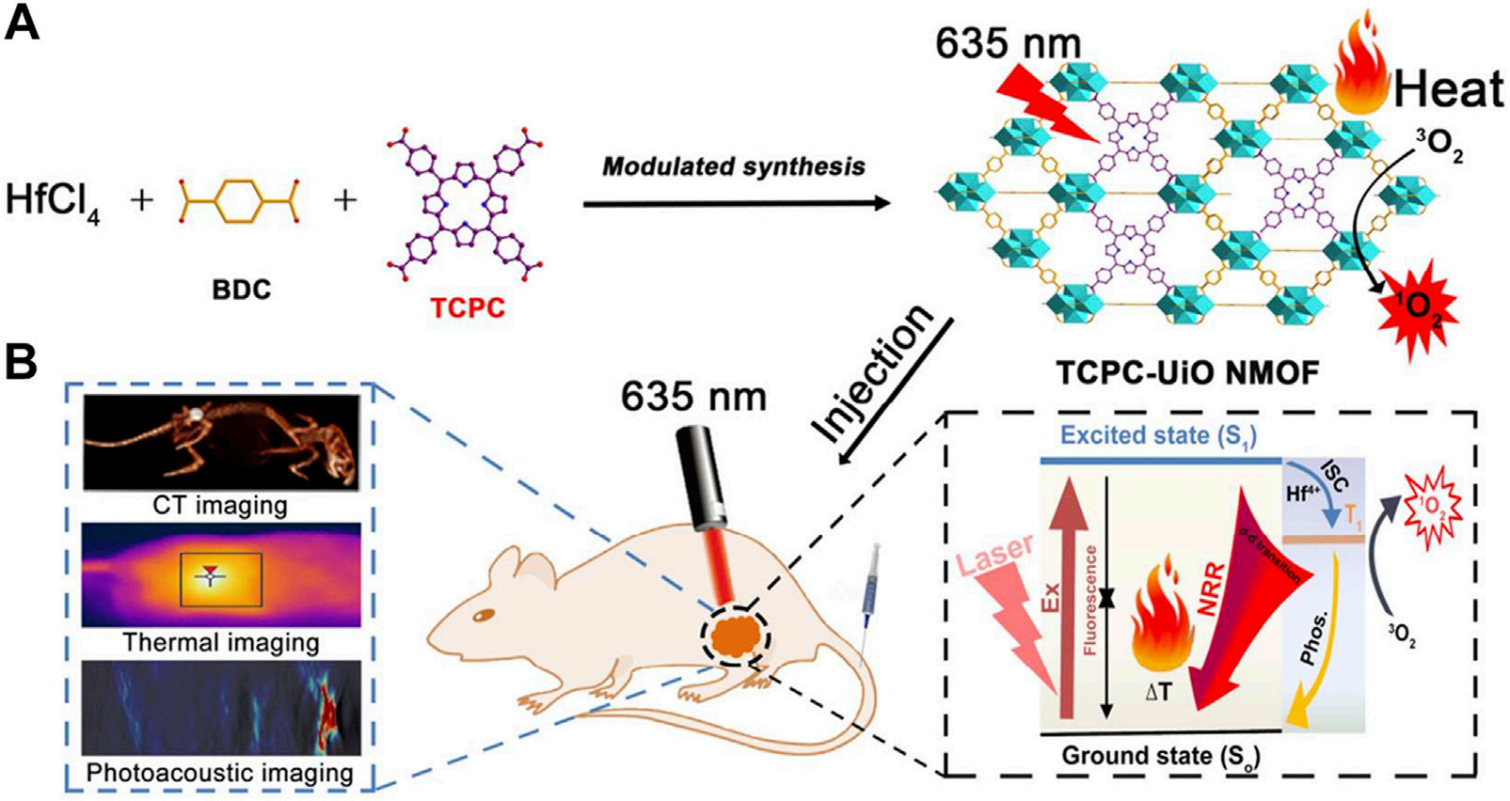
**(A)** Synthesis and **(B)** biomedical application of TCPC-UiO by light activation. Reprinted with permission. Copyright 2018 American Chemical Society (Zheng et al., 2018).

Zheng et al. adopted a streamlined approach to successfully incorporate structurally diverse and differently connected photosensitizing tetracarboxy-chlorin (TCPC) ligands into the Hf-UiO-66 framework while preserving the topological integrity of the parent structure (Figure 8A) (Zheng et al., 2018). Unlike conventional porphyrin-nMOFs, where porphyrins are arranged in a uniform periodic fashion and primarily suited for PDT, the newly synthesized TCPC-UiO exhibits dual potential for PDT and photothermal therapy (PTT). The PTT capability of TCPC-UiO displays remarkable tumor suppression efficacy. The heterogeneity of TCPC-UiO endowed it with high photothermal conversion efficiency, excellent photostability, biocompatibility, and strong X-ray absorption characteristics. These features suggest its promise as a platform for multimodal CT, thermal, and PA imaging applications. *In vivo* experiments demonstrated that TCPC-UiO displayed high antitumor efficacy in H22 tumor-bearing mice, achieving a tumor inhibition rate exceeding 90% (Figure 8B). This study innovatively explored the potential link between the heterogeneous characteristics of TCPC-UiO and its phototherapeutic effects. Contrary to the periodic arrangement of porphyrins in conventional porphyrin-based nMOFs aimed at enhancing PDT efficiency, the introduction of TCPC in this work shifted the focus markedly towards photothermal effect-dominated treatment modalities. This nanodiagnostic and therapeutic system overcame the oxygen dependency limitation inherent to PDT. It also introduced additional therapeutic benefits, paving the way for the expansion and optimization of phototherapeutic properties of porphyrin-based nMOFs in the biomedical field.

## 5 Bacteriochlorin-based nMOFs for photodynamic therapy

Porphyrin and chlorin-based nMOFs have demonstrated potential in PDT. However, these materials are constrained by issues such as PS persistence, which leads to photosensitization in healthy tissue. Additionally, limitations include restricted light penetration depth and tumor hypoxia (Fan et al., 2016). As a deeply reduced form of porphyrin and chlorin, the bacteriochlorin molecule offers several key advantages. Its low absorption in the visible light region reduces skin sensitivity under daily light exposure, while its high absorption in the NIR region (700–850 nm) enhances PDT efficacy (Luo et al., 2020; Xian et al., 2023; Zhang et al., 2023c). Moreover, the Type I reaction mechanism ensures effectiveness even in low-oxygen environments. However, bacteriochlorin’s instability to light and oxygen affects its performance in PDT. Utilizing nMOFs as carriers can enhance the stability and PDT efficacy of bacteriochlorin, potentially offering a more optimized therapeutic solution compared to conventional porphyrin or chlorin-based nMOF systems (Luo et al., 2020).

**FIGURE 9:**
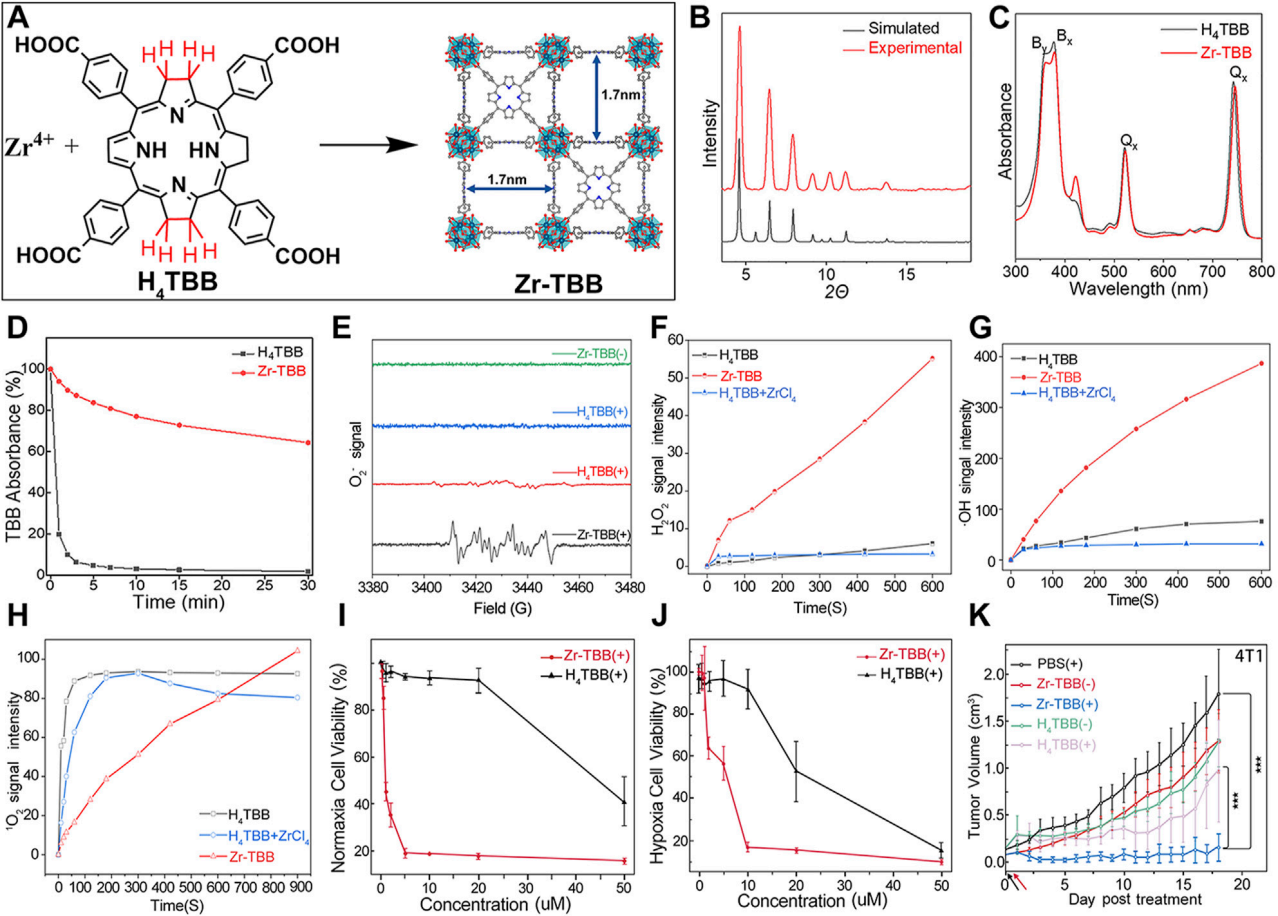
**(A)** Preparation of Zr-TBB nMOFs. PXRD pattern **(B)**, and UV-vis spectra **(C)** of Zr-TBB. **(D)** Time course of H4TBB and Zr-TBB UV-visible absorption in air-equilibrated DMF following light exposure. **(E)** EPR signals indicative of the BMPO adduct formed with superoxide anion (O_2_^−^·). **(F)** Temporal production of H_2_O_2_ using a H_2_O_2_ detection assay. **(G)** Monitoring the time course of hydroxyl radical (·OH) production, as detected through the APF (aminophenyl fluorescein) assay methodology. **(H)** Temporal production of ^1^O_2_ quantified via the SOSG assay technique. Cell viability assessments using MTS tests for Zr-TBB(+) and H_4_TBB(+) under standard oxygen **(I)** and low oxygen **(J)** environments. **(K)** Tumor suppression effectiveness in 4T1 tumor-grafted BALB/c mice following the administration of Zr-TBB and subsequent light exposure. Reprinted with permission. Copyright 2020 American Chemical Society (Luo et al., 2020).

For example, Luo et al. synthesized a novel bacteriochlorin-based nMOF, Zr-TBB, using Zr^4+^ and tetracarboxylic bacteriochlorin (TBB) molecules (Figure 9A) (Luo et al., 2020). Single-crystal X-ray diffraction analysis of Hf-TBB revealed its PCN-224 structure (Figure 9B). UV-Vis testing showed that H_4_TBB exhibits a maximum absorbance peak at 742 nm, nearly ideal for tissue penetration. The molar extinction coefficient values for H_4_TBB’s peak absorption were approximately 12 times higher than those of H_4_TBP (tetracarboxylic porphyrin) and twice those of H_4_TBC (tetracarboxylic chlorin), making H_4_TBB superior as a PS with optimal absorption wavelength and higher ε values. The UV-Vis spectrum of Zr-TBB mirrored the number of peaks found in H_4_TBB, indirectly suggesting its potential as an ideal photosensitizer (Figure 9C). Photostability assessments of H_4_TBB and Zr-TBB in DMF solution at a concentration of 5 μM under 740 nm light (100 mW/cm^2^) revealed that after just 5 min, H_4_TBB’s absorbance at its maximum wavelength dropped below 4% of its initial value, highlighting significant photodegradation (Figure 9D). Conversely, Zr-TBB maintained 73% and 65% of its quantum yield (Qy) peak absorbance after 15 and 30 min of illumination, respectively, demonstrating superior photostability over H_4_TBB. This enhanced stability is attributed to the spatial protection provided by the nMOF structure, which restricts conformational changes in TBB before photo-oxidation occurs. Additionally, the site-isolation properties of Zr-TBB effectively prevent the biodegradation of the TBB ligand. The ability of H_4_TBB and Zr-TBB to generate superoxide anion, hydrogen peroxide, hydroxyl radicals, and singlet oxygen was evaluated using various techniques including electron paramagnetic resonance (EPR), H_2_O_2_ detection kits, aminophenyl fluorescein (APF), and singlet oxygen sensor green (SOSG) (Figures 9E–H). Notably, H_4_TBB displayed efficient singlet oxygen generation capability following a Type II reaction pathway but had lower activity in its Type I mechanism. In contrast, Zr-TBB could not only effectively produce singlet oxygen but also generate other ROS species via the Type I mechanism, such as O_2_^−^·, H_2_O_2_, and ·OH, a feature that confers potential for Zr-TBB in treating hypoxic tumors. Following the establishment of the photochemical characteristics of bacteriochlorin -engineered nMOFs, researchers proceeded to evaluate their antitumor efficacy both *in vitro* and *in vivo*. Cellular experiments demonstrated that Zr-TBB significantly reduced the survival of 4T1 breast cancer cells under both normoxic and hypoxic conditions, confirming the dual Type I and Type II PDT effects of bacteriochlorin (Figures 9I, J). Further *in vivo* studies showed that Zr-TBB combined with light irradiation resulted in a 91% tumor growth inhibition rate and up to a 40% tumor regression rate in a 4T1 tumor-bearing mouse model, highlighting its potential as an anticancer treatment strategy (Figure 9K). The combination of bacteriochlorin with nMOFs not only further expands the phototherapy applications of porphyrin derivatives but also offers new possibilities for the use of photo-unstable molecules.

**FIGURE 10:**
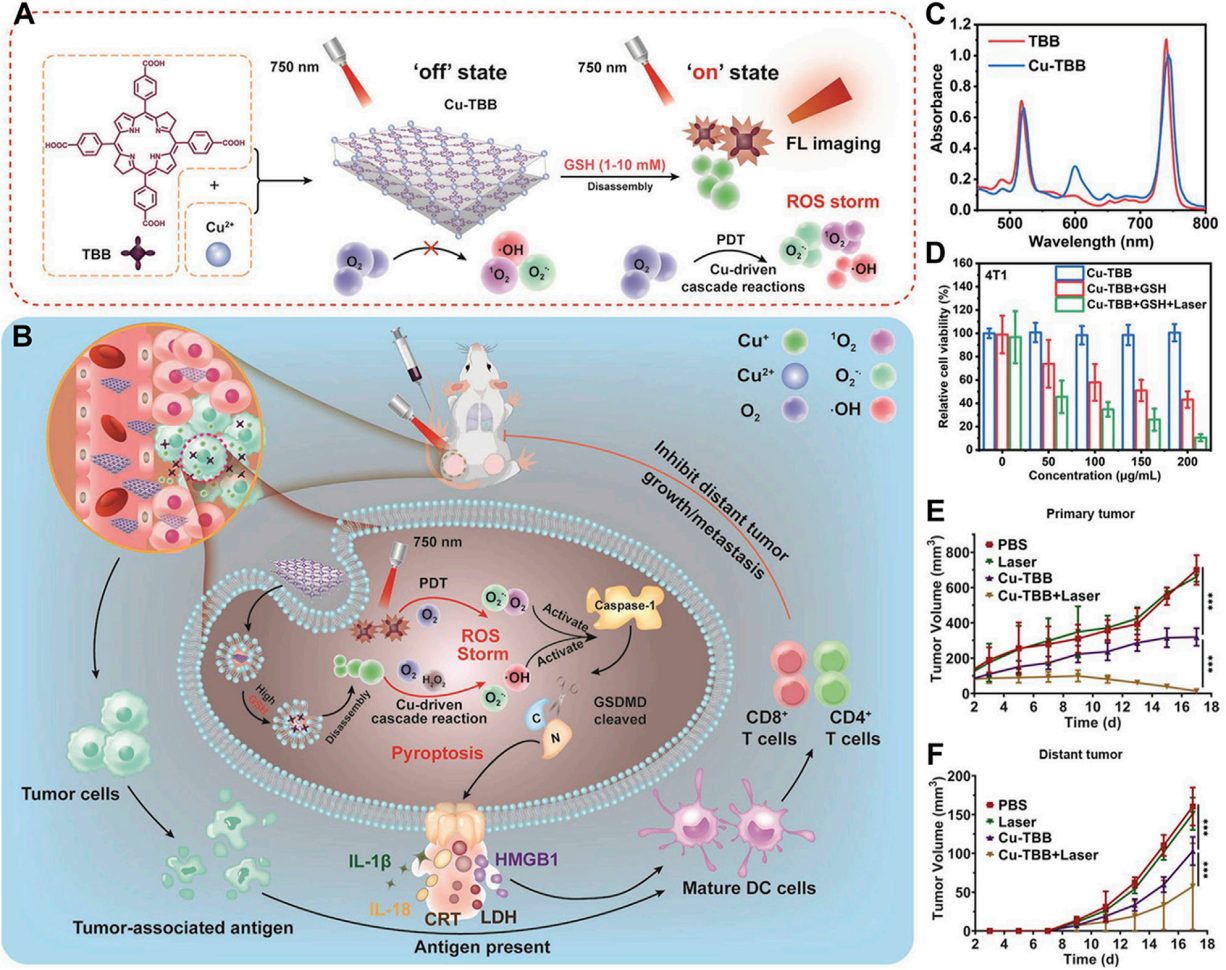
**(A)** Diagrammatic representation of the fabrication process and **(B)** the anti-neoplastic rationale underlying the Cu-TBB nanosheet formulation. **(C)** Comparative UV-vis spectral data for TBB and its Cu-TBB nanosheet derivative. **(D)** Assessment of 4T1 cell viability in response to treatment regimens. **(E)** Plotting the temporal evolution of primary tumor volumes following intervention. **(F)** Graphical depiction of the growth kinetics for distant tumors over time. Reprinted with permission. Copyright 2023 John Wiley & Sons, Inc (Zhang et al., 2023c).

Beyond coordination with Zr^4+^, which itself lacks biological function, bacteriochlorin can also coordinate with Cu^2+^. Redox-active Cu-dependent enzymes serve as cofactors in various biological processes (Zhang et al., 2023c). There is evidence that GSH can mediate the reduction of Cu^2+^ to redox-active Cu^+^ species, which in turn catalyzes molecular O_2_ into superoxide anion (O_2_^−^·). Furthermore, Cu^2+^-induced depletion of GSH has been shown to exacerbate oxidative stress in cancer cells, thereby enhancing its antitumor effect. Coordination of bacteriochlorin with Cu^2+^ not only amplifies the oxidative stress induced by ROS but also overcomes hypoxia’s limitation on PDT efficacy, harnessing the immunogenic effects of PDT to activate the immune system and eradicate distant tumors (Zhang et al., 2023c). As demonstrated by Zhang et al., Cu-TBB nanosheets were synthesized through coordination of Cu^2+^ with tetracarboxylic bacteriochlorin (Figure 10A) (Zhang et al., 2023c). It was confirmed that Cu-TBB could be activated in a GSH-rich tumor microenvironment, releasing Cu^+^ and TBB. Uniquely, the released Cu^+^ catalyzed a series of reactions, generating O_2_^−^· and highly destructive ·OH. Concurrently, upon irradiation with 750 nm laser light, TBB generated ROS, including O_2_^−^· and ^1^O_2_. Encouragingly, the pyroptosis triggered by the synergy of Cu^+^-catalyzed reactions and PDT cooperatively eradicated primary tumors. Alongside this, the maturation of dendritic cells and activation of T-cells effectively controlled the development and metastasis of distant tumors (Figure 10B). UV-Vis spectroscopy revealed that MOF materials derived from bacteriochlorin maintained strong light absorption at 730 nm, with Cu-TBB exhibiting a slight red shift compared to TBB (Figure 10C). After verifying that the absorption of bacteriochlorin molecules was not significantly diminished, the authors proceeded with cytotoxicity validation tests. Specifically, the authors employed the MTT assay, a commonly used method (Wang et al., 2018; Cai et al., 2019; Ling et al., 2019; Chen et al., 2020; Li et al., 2020; Zhu et al., 2020), to evaluate the cytotoxicity of Cu-TBB nanomaterials. MTT assay results indicated that Cu-TBB exhibited negligible toxicity toward 4T1 cells in the absence of additional conditions, highlighting its excellent biocompatibility (Figure 10D). However, in the presence of GSH (10 mM), cell viability significantly decreased due to ROS generated by Cu^2+^ catalysis. More strikingly, when combined with 750 nm laser irradiation, cell death reached approximately 90% due to the surge in ROS levels (Figure 10D). To assess the photodynamic immunotherapy efficacy of Cu-TBB, researchers employed a bilateral 4T1 tumor BALB/c mouse model. Primary tumors were established by inoculating 4T1 cells in the right inguinal region, followed 3 days later by inoculation of distant tumors in the left inguinal region, with continuous monitoring of tumor growth at both sites. A pivotal finding was that tumors in the Cu-TBB + Laser group were virtually eliminated (Figure 10E). Cu-TBB exerted a strong control over distant tumors, significantly reducing tumor volume and demonstrating remarkable efficacy (Figure 10F).

This study highlights that bacteriochlorin-based nMOFs can be precisely activated in the tumor microenvironment when used in conjunction with bioactive metals. Leveraging their potent ROS generation capacity, these nMOFs induce pyroptosis and clear primary tumors. Additionally, they boost tumor immunogenicity. This further stimulates T-cell-dependent adaptive immune responses, effectively curtailing the progression of distant tumors while minimizing non-specific damage to the body. This achievement not only advances the design concept of innovative nanostructures based on bacteriochlorin nMOFs but also paves the way for exploring tumor microenvironment-responsive porphyrin derivatives in the realm of specific cancer immunotherapy PDT nanomaterials.

## 6 Summary of photophysical properties comparison: porphyrins, chlorins, bacteriochlorins, and their corresponding nMOFs

**FIGURE 11:**
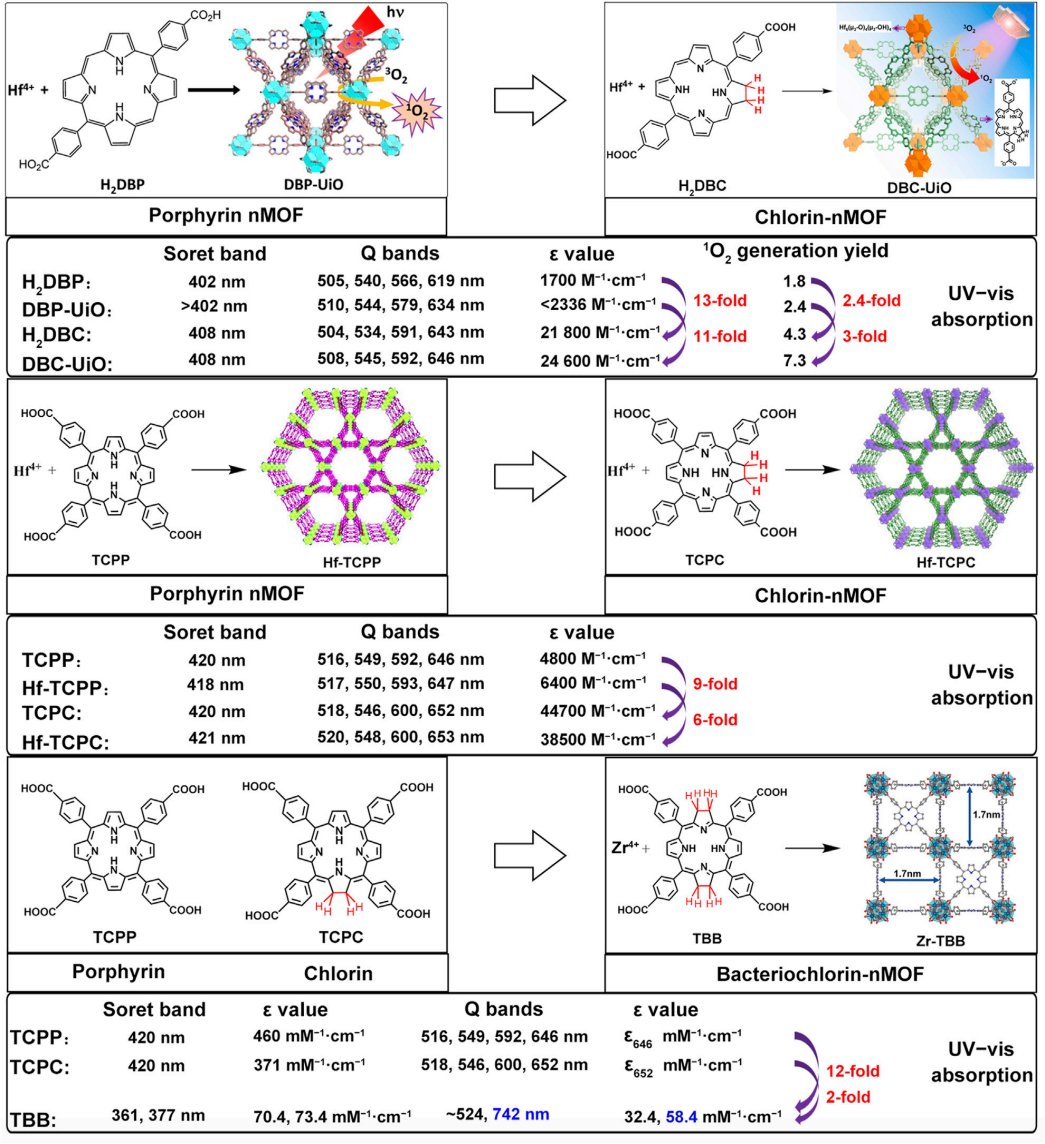
Summary of photophysical properties comparison: from porphyrins, chlorins, to bacteriochlorins, and their nMOFs.

To better distinguish the structural design improvements from porphyrin to chlorin and then to bacteriochlorin molecules, we summarized their impact on PDT. We focused on the photochemical properties of the corresponding nanomaterials, examining the transitions from porphyrin, chlorin to bacteriochlorin and from porphyrin-nMOFs, chlorin-nMOFs to bacteriochlorin-nMOFs (Lu et al., 2015; Lu et al., 2016; Luo et al., 2020). As shown in Figure 11, neither the porphyrin-to-chlorin transition nor the shift from porphyrin-nMOFs to chlorin-nMOFs significantly altered the ultraviolet absorption profiles, including the characteristic Soret band and four Q bands. However, there was a dramatic increase in the molar extinction coefficient at the maximum absorption wavelength. For instance, moving from porphyrin (H_2_DBP) to chlorin (H_2_DBC), the molar extinction coefficient at the maximum absorption wavelength (ε value) increased from 1,700 M^−1^ cm^−1^ to 21,800 M^−1^ cm^−1^, enhancing by approximately 13 times. This increase in absorption intensity is hypothesized to be due to enhanced overlap between HOMO and LUMO orbital electron clouds, leading to greater transition dipole moments and consequently higher oscillator strengths, thus increasing the molar absorptivity (Petit et al., 2006; Dong et al., 2012; Wang and Kitao, 2012; Narra et al., 2015). Similarly, the ε value for DBP-UiO nMOF to DBC-UiO nMOF transition increased by about 11 times. These findings indicate that transitioning from porphyrin to chlorin molecules significantly enhances photon capture capability. For photosensitizers, this means stronger photoreactivity under identical lighting conditions, resulting in higher singlet oxygen quantum yields and thus stronger PDT effects. Enhanced PDT can achieve equivalent therapeutic outcomes with lower dosing, thereby mitigating potential toxic side effects. Literature reviews show that the ε values at the maximum absorption wavelengths increased by 9 times for TCPP to TCPC (Lu et al., 2016). Additionally, for Hf-TCPP nMOF to Hf-TCPC nMOF, these values increased by 6 times, laying the foundation for improved PDT efficacy (Figure 11).

Furthermore, UV testing revealed that the maximum absorption wavelengths for porphyrin and chlorin molecules were 646 nm and 652 nm, respectively, while that of bacteriochlorin (TBB) was 742 nm, falling within the NIR range. This phenomenon may be due to a red shift caused by a narrowing of the bandgap, which makes electron transitions easier and enhances the material’s excitability for photodynamic therapy (Jiang et al., 2018). Compared to TCPP and TCPC, the TBB molecule exhibits only two absorption peaks in the Q bands. The reduction in the number of absorption peaks fundamentally stems from the transformation of the porphyrin pyrrole rings from rigid to flexible due to the reduction of double bonds. This leads to an overlap of the molecular absorption transition levels, resulting in fewer absorption peaks. This longer wavelength is more favorable for tissue penetration during illumination. Compared to TCPP and TCPC molecules, the ε values at the maximum absorption wavelength of TBB increased by 12 and 2 times, respectively (Figure 11). These comparative results suggest that transitioning from porphyrins, chlorins to bacteriochlorin, may significantly enhance PDT efficacy.

**FIGURE 12:**
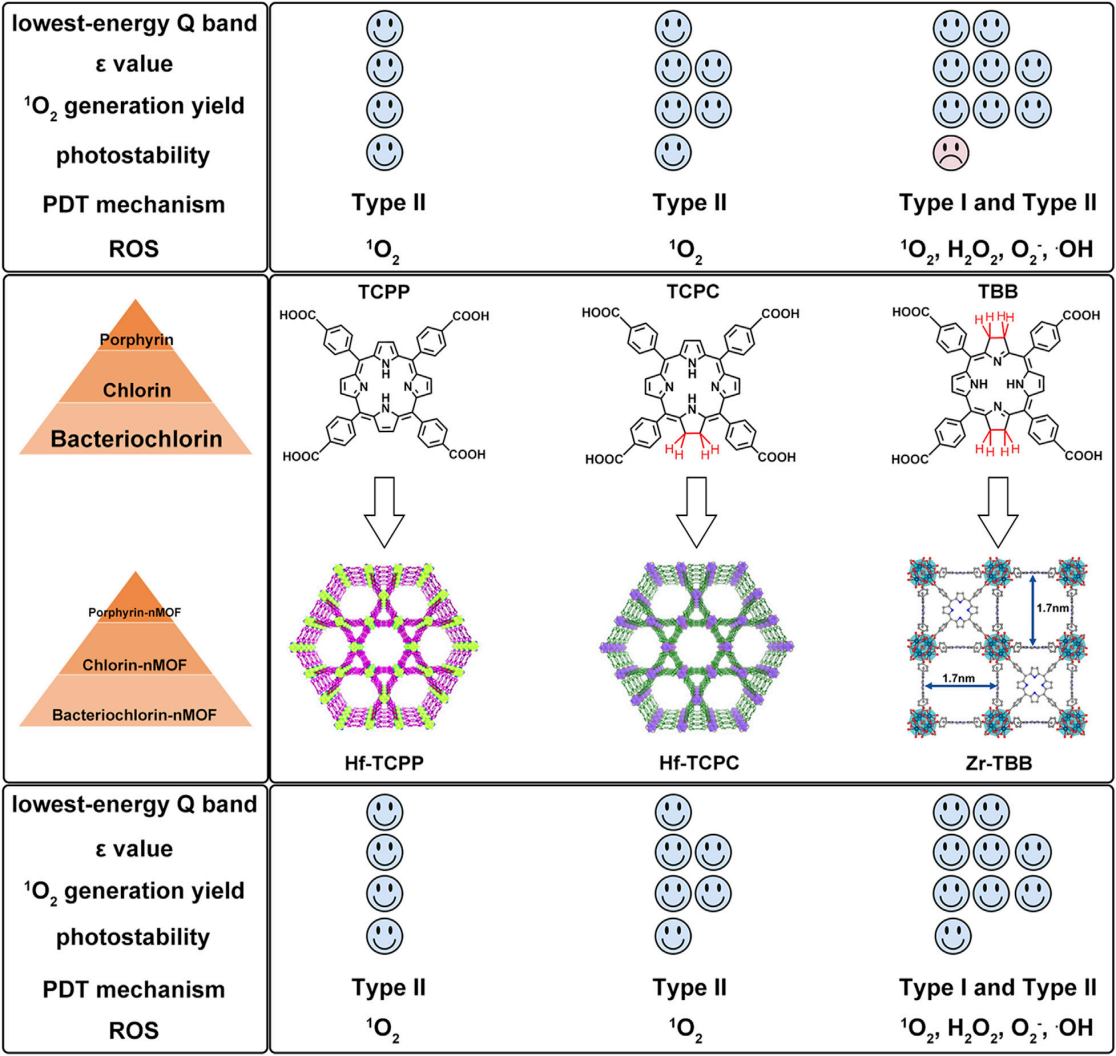
Comparison of advantages and disadvantages of porphyrins, chlorins, bacteriochlorins, and their corresponding nMOFs.

To further highlight the enhanced PDT effects from porphyrins, chlorins to bacteriochlorins, as well as their corresponding nMOFs, we conducted comparative analyses of their photochemical properties. The transition from porphyrins to chlorins involves the reduction of one double bond, resulting in minor shifts in the maximum absorption wavelength (Lu et al., 2016). This transition significantly increases the ε values and singlet oxygen quantum yields. Photostability remains largely unchanged. Transitioning from porphyrins to bacteriochlorins involves the reduction of two double bonds, leading to a red shift of nearly 90 nm in the maximum absorption wavelength, with further enhancement of ε values and singlet oxygen quantum yields compared to chlorins (Luo et al., 2020). These findings clearly demonstrate the advantages of chlorins and bacteriochlorins over porphyrins (Figure 12). However, experimental results indicate that synthesized bacteriochlorin is more prone to oxidation in oxygen-rich environments due to structural changes from rigid to flexible. This increased susceptibility to oxidation may affect its repeated use for PDT. Surprisingly, encapsulating bacteriochlorin into nMOFs significantly improves its photostability. For example, Zr-TBB nMOFs prepared from TBB enhance TBB stability due to spatial constraints within the nMOFs framework. These constraints prevent structural changes before photocatalytic oxidation occurs, thus avoiding decomposition. Additionally, under anaerobic conditions, both Zr-TBB and H_4_TBB exhibit good photostability. While porphyrins and chlorins rely on type II photochemical mechanisms dependent on oxygen to generate singlet oxygen for PDT treatment, bacteriochlorin can engage in both type I and type II PDT mechanisms. This indicates that even in hypoxic tumor microenvironments, bacteriochlorin and its nMOFs can still be effective for PDT. They remain effective without being severely limited by poor photostability (Figure 12). Not only do nMOFs enhance the photostability of bacteriochlorin; we also speculate that molecular self-assembly systems possess the potential to further augment its photostability. Moreover, leveraging the instability of bacteriochlorin might avoid the photosensitivity issues associated with residual photosensitizers in skin and diseased areas. Additionally, the degradable nature of these frameworks could be utilized to carry chemotherapeutic drugs and immunotherapies for desired light-controlled drug release. This could further inhibit metastatic or drug-resistant tumors, pointing to new directions for future research.

## 7 Conclusion and perspectives

Over the past few years, PDT based on porphyrins has gained significant attention for its potential in cancer treatment (Freund et al., 2021; Pham et al., 2021). However, traditional porphyrin-based PSs face several challenges: hydrophobic aggregation, hypoxia within tumors, and limited light penetration due to absorption in the shorter wavelengths. To address these issues, researchers have developed various strategies, such as the design and synthesis of porphyrin-based porphysomes (Huynh and Zheng, 2014; Jin et al., 2014; MacDonald et al., 2014), COFs linked by porphyrins, and MOFs integrated with porphyrins (Ding et al., 2022; Sun et al., 2023). To overcome the limitation of light penetration, efforts have focused on developing PSs that can be activated by NIR light (Fang et al., 2022), utilizing two-photon absorption (Huang et al., 2016; Shen et al., 2016; Zhang et al., 2019; Soleimany et al., 2024) or upconversion NPs (Chen et al., 2023a; Nsubuga et al., 2024) to indirectly activate conventional PSs. These approaches enhance light penetration depth, enabling the effective treatment of deep-seated tumors (Zhang et al., 2023b). For hypoxic tumors, strategies such as direct oxygen supply (Wei et al., 2021), using catalysts to generate oxygen from hydrogen peroxide, or employing mitochondrial respiratory inhibitors to reduce oxygen consumption have been employed to improve the tumor microenvironment. These methods help enhance the efficacy of PDT. While initial approaches may advocate for complex adjunctive strategies in conjunction with PSs, a more refined solution lies in the strategic modification of the photosensitizer’s molecular structure. By doing so, we can effectively tackle the challenges associated with excitation wavelength penetration and hypoxic environments, thereby achieving a significant and simplified amplification of PDT efficacy (Lu et al., 2015). For instance, reducing porphyrins to chlorins or bacteriochlorins improves their therapeutic outcomes. Chlorins exhibit a ∼10-fold increase in molar extinction coefficient at their maximum absorption wavelength (∼650 nm), which not only boosts photon utilization but also reduces the required drug dose (Lu et al., 2016). Bacteriochlorins have a red-shifted maximum absorption wavelength of approximately 740 nm and a molar extinction coefficient that is more than 12 times higher than that of porphyrins. They can undergo both oxygen-dependent type II and non-oxygen-dependent type I photodynamic processes. These processes generate ROS that are effective in treating hypoxic tumors (Luo et al., 2020).

As an emerging class of porous materials, MOFs can prevent the aggregation of photosensitizers and offer new possibilities for their application (Sun et al., 2023). In particular, porphyrin-nMOFs have shown great potential in tumor PDT (Ding et al., 2022). These hybrid materials can enhance the photophysical properties of porphyrins, improve their delivery efficiency, and enhance biocompatibility (Fu et al., 2022). This review explored the characteristics of MOFs prepared from porphyrins, chlorins, and bacteriochlorins, including their crystal structures and ROS generation capabilities. Moreover, this review highlighted how molecular design can enhance PDT mechanisms and outcomes, particularly focusing on applications that overcome tumor hypoxia and metastasis. Optimizing the design of porphyrin-nMOFs could advance research in the PDT field and facilitate clinical translation. However, rapid development also brings new requirements. Despite the promising results with porphyrin-nMOFs in PDT applications, several scientific questions and technical challenges remain: 1) For primary tumors located deep within the body, such as liver or lung cancers, traditional light sources for PDT are limited by light penetration depth. Therefore, alternative activation methods like X-ray radiation, ultrasound, and magnetic fields, which have better penetration through human tissue, could be considered. 2) Given the short lifetime and limited range of ROS, developing targeted photosensitizer systems that can specifically deliver to subcellular compartments, such as mitochondria, lysosomes, or nuclei, can enhance the efficacy of PDT. 3) To address tumor metastasis or effectively eliminate distant metastatic cancer cells, immunotherapy is a valuable adjunct, as it can activate the immune system to target residual cancer cells. The synergistic use of chlorin or bacteriochlorin-nMOFs with immunotherapy may significantly enhance comprehensive cancer treatment. We believe that by prioritizing and proposing well-reasoned research strategies to address these issues, the application of nMOFs prepared from porphyrins and their derivatives in the biomedical field can be further advanced.
